# The blurry lines between popular media and party propaganda: China’s convergence culture through a linguistic lens

**DOI:** 10.1371/journal.pone.0297499

**Published:** 2024-01-25

**Authors:** Jun Lang, Zhuo Jing-Schmidt

**Affiliations:** 1 Asian Languages and Literatures Department, Pomona College, Claremont, CA, United States of America; 2 Department of East Asian Languages and Literatures, University of Oregon, Eugene, Oregon, United States of America; Zhejiang Gongshang University, CHINA

## Abstract

There is a growing body of scholarly evidence that media convergence blurs the boundary between media production and media consumption and obscures the lines between institutions and individuals. Media convergence in the context of China has garnered attention in communication studies and in cultural studies. However, there is a scarcity of research on convergence culture from a linguistic perspective. Recent research has generated initial evidence that state media appropriates a pop-cultural social address for clickbait and information management in China’s digital media space. However, the patterns and perceptual reality of linguistic convergence remain unexplored. This study investigates popular and party uses of *xiaojiejie* ‘little older sister’, a familiar expression of fictive kinship reborn as a viral personal reference and social address in China’s convergence culture. Analysis of the Target Group Index in the Baidu search engine suggests *xiaojiejie* is gaining ground over its predecessor among young Chinese. Trends analysis of its usage in WeChat public accounts showed that the term has spread from popular media to state media, which employs the viral address to drive clickbait and disguise propaganda. An online survey of young Chinese WeChat users (*N*=330) on their perception of *xiaojiejie* headlines from WeChat public accounts showed that respondents could not tell state media uses from popular uses, providing perceptual evidence of the blurry boundaries between popular and state media uses of the viral address. The findings demonstrate the reality of linguistic convergence driven by participatory performance and its perceptual consequences in China’s convergence culture.

## Introduction

### Convergence culture as a theoretical framework

There is a growing body of scholarly evidence that media convergence blurs the boundary between media production and media consumption and obscures the lines between institutions and individuals [1–9]. The ubiquity of intermediality is a hallmark of convergence culture, which Jenkins [4] describes as a participatory culture where ‘old and new media collide, where grassroots and corporate media intersect, where the power of the media producer and the power of the media consumer interact in unpredictable ways’. Jenkins views convergence culture as constitutive of contemporary popular culture driven by unprecedented technological innovations, industrial shifts, as well as radical sociocultural and economic transformations. The concept of convergence culture, notwithstanding its romanticization of media convergence as a unifying force of democratic mobilization, as critics point out [10, 11], captures the complex intertwinement and dynamic interaction between top-down media design and bottom-up participation in media production and consumption, which has become our everyday lived experience in the age of perpetual connectivity and global digitization of human communication [12]. As such, the concept offers a theoretical framework within which to probe local contexts and culture-specific forces that shape media convergence and participatory culture, which in turn enrich and refine the theoretical construct, as can be seen in recent advancements in transmedia studies [13] and comparative media studies [14].

In the context of China where Internet users had exceeded 1.05 billion by June 2022, accounting for 74.4% of the country’s total population [15], media convergence has expanded rapidly and systematically under the government’s Internet Plus and China 2025 policies [16, 17]. China boasts an “intelligent, ecological, and flexible” ecosystem of media convergence that encompasses infrastructure, content, and governance [18]. Chinese society has become a “platform society” in the sense that actors from market, state, and the private sphere are interdependent, overlapping and interacting in complex ways that obscure the distinctions between the private and the public, as defined by van Dijck, Poell, and de Waal [19]. China’s convergence culture exemplifies the “articulation of institutional and cultural processes of platformization” [20], and defies simple dichotomies of grassroots activism versus state control [21].

Media convergence in the context of China has garnered intense attention in media and communication studies where findings suggest a changing dynamic in the interaction between state media, popular media, and the public [17, 22–25]. Su [23] showed how Chinese political communication has undergone a progressive shift away from a top-down control model to a state–society negotiation model as a result of media convergence. Li, Gong and Mou described how small niche-player media agilely pioneered the process of media convergence and innovation in China’s lifestyle media production within the constraints of macro-political forces [26]. Duan showed that clear-cut boundaries that previously demarcated media channels have dissolved in China’s digital mediascape [17]. More recently, convergence culture has entered the theoretical horizon of Chinese cultural studies [27]. Qu [28] demonstrated how a state-sponsored documentary showcasing Chinese culinary superiority and social prosperity strategically tied to national identity took on a life of its own in the ecology of China’s convergence culture, transcending its ideological designation and thriving at the intersection of media convergence and participatory culture.

While convergence culture has been productively applied in accounting for new modes of Chinese political communication, new forms of media innovation, and new artefacts of national identity construction, there is a scarcity of research on China’s convergence culture from a linguistic angle. An exception is Lang and Jing-Schmidt [29], a sociolinguistic study on the uses of the viral social address *meinü* ‘beautiful woman’ in popular culture and party propaganda. The researchers conducted an online survey of the usage and perception of the address term across social categories. They found a bottom-up diffusion of pop-cultural uses of the term into the digital platforms of state media and argued that state media speaks the language of pop culture to be relatable, drive traffic, and attract clickbait for strategic information control and enhanced propaganda. Their findings highlighted the sociolinguistic manifestations of China’s media convergence and participatory culture and raised questions about the place and the role of language in media culture, an area of research that is underrepresented both in the literature on convergence culture and in Internet linguistics.

In their online survey, Lang and Jing-Schmidt [29] found a generational variation in the popularity of the social address *meinü*, opposite to the age-related trend found in earlier studies. The younger group, aged 18–29, was significantly more likely to say that they never or rarely used *meinü* compared to the 40–49 and 50 and above groups who were significantly more likely to report often or always using the term. In other words, while the older generation continues to embrace *meinü*, the younger generation has shifted their interest to the next shiny new thing in social address. From a variationist sociolinguistic perspective, their findings indicate a downward trend of the popularity of *meinü*. The researchers also reported that young female respondents in their study found *meinü* to be outdated and corny and some shared that a new, chic alternative was *xiaojiejie* (小姐姐) ‘little older sister’. Thus, there is anecdotal evidence that *xiaojiejie* is replacing *meinü*. However, this new phenomenon with its sociolinguistic detail remains unexplored. But who and what is *xiaojiejie*? Where does it come from?

#### Xiaojiejie—A new social address

**Fig 1:**
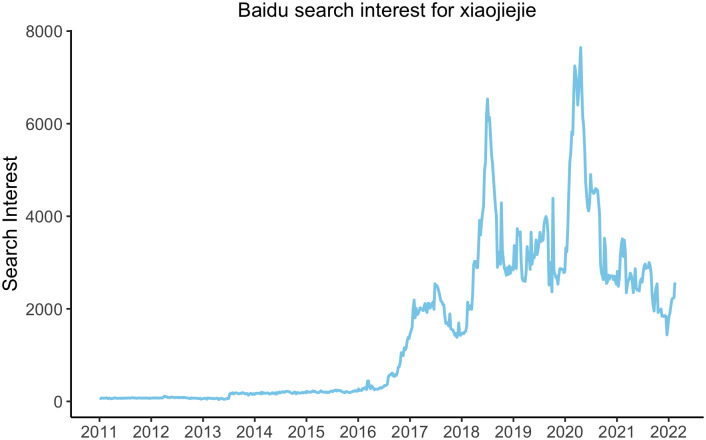
Trajectory of Baidu search interest in xiaojiejie from 2011 to 2022. Data obtained from Baidu Index in April 2022.

*Xiaojiejie* is not new. It is a noun phrase consisting of two parts, the modifier *xiăo* ‘little, small’ and the head noun *jiĕjie*, an endearing reduplication of the kinship term *jiĕ* ‘older sister’. There is nothing remarkable about its conventional use, which typically occurs in a social encounter involving two or more youngsters, one of whom is female and slightly older than the other(s). The younger one(s) will be instructed by an adult to call the older girl *xiaojiejie* in a fashion of “fictive kinship” [30, 31]. The new identity of this expression as a popular social address in the digital age has its origin in contemporary teenage fan culture and can be traced back to the enchantment of Chinese fans by the nine-member teenage idol group *μ*’s (Muse) of the Japanese multimedia franchise Love Live! (https://love-live.fandom.com/) whose 2016 Fan Meeting Tour in Shanghai popularized *xiaojiejie* among Chinese fans who used it admiringly for the members of the idol group [31]. Apparently, following the 2016 fan event, *xiaojiejie* went viral in Chinese cyberspace, as can be seen in Fig 1 with the abrupt surge of search interest in China’s search engine Baidu. Search interest in *xiaojiejie* had been practically nonexistent before 2016 but trended up sharply later that year and reached a first peak in early 2017, followed by more frequent and higher peaks in subsequent years.

The referential reach of this popular term quickly widened from the fantastic world of teenage fandom to the social space of real people. All of a sudden, the girlfriend of a neighbor’s nephew was welcomed to the family’s WeChat group as a *xiaojiejie*! In a personal way like that, Chinese social media came to witness the mesmerizing arrival of a new social address in real time. Unsurprisingly, *xiaojiejie* enjoys a meme-like quality in Chinese social media. A casual keyword search for *xiaojiejie* in the stickers section of the mobile phone interface of China’s “super app” WeChat returned dozens of digital stickers of *xiaojiejie* images with a cuteness overload, if overtly flirtatious. Some of these stickers carry captions in which *xiaojiejie* is used in blunt requests for the WeChat handle of the cute addressee or otherwise in what can be described as vulgar “virtual catcalls” [29]. The meme-like quality of the digital stickers suggests the popularity and spreadability of *xiaojiejie* beyond popular fandom and its viral exuberance in China’s meme-filled participatory culture.

Unlike *meinü* that is semantically associated with female physical attractiveness, *xiaojiejie* lexically meshes the gender aesthetics of the Japanese kawaii culture of cute femininity pervasive in East Asian popular culture [32, 33] and familiarity and endearment of Chinese fictive kinship [30]. If cuteness in language is socially indexical of a chic, playful, and youthful identity, as Shi and Jing-Schmidt [34] argued, the popular use of *xiaojiejie* as a social address no doubt confers the language user a trendy, youthful, bubbly, and flirtatious style. The sense of familiarity associated with fictive kinship makes the user of this address disarmingly cuddly and endearing. These lexical semantic features may explain why *xiaojiejie* is gaining popularity and may be replacing *meinü* among certain demographics. The apparently ongoing popularization of *xiaojiejie* as a social address provides an ideal case study of linguistic convergence as an emergent process.

Studies of China’s convergence culture have so far primarily focused on the convergence environment of Chinese digital media and the interaction between media stakeholders. No research has approached convergence culture by asking whether and to what extent the blurry lines between institutional and popular media production are perceptually real to media users. Yet, media products exist in the perceptual world of those who use them. Therefore, analysis of media convergence must account for the user perspective. There is now a growing body of media research that argues for treating user and user perception as analytical concepts, stressing the importance of understanding user perception of media landscapes where traditional and digital media are blended whereby users actively participate in the production and consumption of media in a convergence culture [35–38]. However, existing research on user perception of hybrid media production tends to focus on users in Europe and the United States [38, 39]. User perception of media convergence in the context of China remains poorly understood.

Given the initial evidence from Lang and Jing-Schmidt’s [29] sociolinguistic survey and corpus analysis of a linguistic and stylistic convergence in China’s digital media, a user-oriented analysis of this convergence is needed to test its perceptual reality. If linguistic convergence can be empirically shown to have perceptual reality, as, for example, when the public fails to recognize party propaganda dressed as popular gimmick, then that reality not only has consequences for understanding the motivation and performance of the relevant social actors, it also raises pressing questions about political cognition and public trust. Proper answers to such questions are important for a deeper analysis of China’s convergence culture and demand that the research gap in understanding user perception of the boundaries between China’s popular and party media be filled. It is the goal of this study to fill that gap.

#### This study

Following Lang and Jing-Schmidt [29], this study further explores the blurry linguistic and stylistic boundaries in China’s convergence culture through the lens of changing social address as a barometer of sociocultural transformations. We do so with a particular focus on the perceptual reality of linguistic convergence as a vital but overlooked part of China’s convergence culture.

Specifically, we ask three questions that incrementally address this research focus:

1. Is there evidence of emergent popularity of *xiaojiejie* vis-a-vis *meinü*?
2. If so, is there evidence that *xiaojiejie* is spreading from popular to state media?
3. If so, is there perceptual evidence of blurring boundaries between popular and state media in their usages of *xiaojiejie*?

Based on Lang and Jing-Schmidt’s [29] findings that showed the waning popularity of *meinü* among younger Chinese and the foregoing observation of the abrupt uptick of search interests in *xiaojiejie* on Baidu, our hypothesis regarding the first research question is:

Hypothesis I: *Meinü* is losing ground to *xiaojiejie* among younger Chinese.

Based on the theory of convergence culture and given the pop-cultural origin of *xiaojiejie*, if Hypothesis I is confirmed, our hypothesis regarding the second research question would be:

Hypothesis II: *Xiaojiejie* has spread from popular media to state media in a process of lexical convergence.

Based on the entailment of convergence culture that convergence is a boundary-blurring process, if the bottom-up diffusion of *xiaojiejie* or Hypothesis II is confirmed, our hypothesis regarding research question 3 would be:

Hypothesis III: The blurring boundaries between popular and state media in their uses of *xiaojiejie* have a perceptual reality.

To test these hypotheses, we utilize a combination of digital media data and perceptual data from an online survey. As our findings will show, popular media usage predated state media usage of *xiaojiejie*, but subsequently the two converged in frequency trends. State media appropriated popular usage specifically for applauding female paragons in positive energy discourse. Survey respondents generally could not tell state media uses from popular media uses of *xiaojiejie*, providing evidence of a perceptually real linguistic convergence between popular and party media uses of the term.

## Data and methods

### Baidu Index data for testing hypothesis I

Age is a sociolinguistic variable in the sense that sociolinguistic variations across age groups can reveal tendencies of language change, typically driven by younger generations [40, 41]. To test Hypothesis I, we conducted secondary analyses on Baidu Search Interests in *meinü* and *xiaojiejie* across age groups in Baidu Index. Baidu is the leading search engine in China and Baidu Index provides the search trends of selected keywords, in the same way Google Trends indicates public information needs and issue salience [42]. Specifically, we compared *meinü* and *xiaojiejie* by their Target Group Index (TGI), which measures search interest across age groups [43]. The higher the TGI score, the greater the interest of a target group in the search object. Hypothesis I predicts a strong association between TGI and age: the younger age groups will show a higher TGI for *xiaojiejie* than for *meinü* whereas the older age groups will show a higher TGI for *meinü* than for *xiaojiejie*. A chi-square of independence was computed on the association between TGI and age group.

### Headlines from WeChat public accounts for testing hypothesis II

To test Hypothesis II, we traced the frequency trends of *xiaojiejie* over regular time intervals starting from its first occurrence as a popular social address or personal reference. To this end, we conducted a keyword search for *xiaojiejie* in WeChat public accounts on the smartphone interface in January 2022 and collected all the headlines returned from the search that contained *xiaojiejie*. The rationale for this data selection was fourfold:

1. We chose WeChat for the analysis because of its ubiquity in China and its dominance in Chinese daily life. With over 1.3 billion monthly active users at the end of March 2023, this multi-functional social media platform permeates every aspect of Chinese life and is known as China’s “super app”. As such it offers an ideal platform for observing linguistic innovation and media convergence.
2. WeChat public accounts (公众号) where individuals and organizations publish content, provide service, and attract followers are at the forefront of China’s media convergence. In this media ecosystem, savvy influencers and party newspapers alike compete for eyeballs traditional media cannot reach [44].
3. The smartphone interface was chosen because of the nearly absolute penetration and indispensability of mobile phones for daily life in China. The proportion of China’s Internet users using mobile phones to access the Internet was 99.6% by June 2022 [15].
4. Headlines were chosen for the analysis for two reasons. First, headlines are crafted to catch attention, which has become the biggest commodity in social media and the new currency of platform economy [45]. Second, to arouse curiosity and grab attention, WeChat headlines that use *xiaojiejie* most likely do so for its newly acquired digital pop-cultural meaning and its social indexical function, which are at the heart of the present study.

To understand the emergence and diffusion of *xiaojiejie* across types of media, we manually annotated and sorted the WeChat public accounts that used *xiaojiejie* in their headlines into two types: state media and popular media. State media includes official public accounts of party-controlled flagship media outlets such as People’s Web, People’s Daily, China Central Television (CCTV) Web, CCTV News, Xinhua News Agency, among others. Popular media includes what is known as self-media accounts (自媒体) and marketing accounts (营销账号). The frequency trajectories of *xiaojiejie* in the headlines from the two types of accounts were visualized as annualized time series in *R* (4.2.1).

Additionally, we built two corpora to identify distinctive linguistic features and themes in headlines containing *xiaojiejie* across the two types of media. One corpus included headlines from WeChat public accounts of state media, whereas the other comprised headlines from WeChat public accounts of popular media. We then compared the lexical semantic, stylistic, and thematic features of the headlines in these two corpora using *R*. By examining the frequencies of words and punctuation marks used in each corpus, we aimed to pinpoint the most salient lexical semantic and stylistic features and recurring themes across the two media types.

### Qualtrics survey on perception for testing hypothesis III

To test hypothesis III, we designed an anonymous online survey on Qualtrics (see S1 Appendix). The survey collected demographic information (Age and Gender) and background information including 1) Frequency of WeChat use, 2) Familiarity with WeChat public accounts, 3) Familiarity with *xiaojiejie*, and 4) Understanding of who *xiaojiejie* is used to address or refer to. The main task of the survey was to ask respondents to identify the media source of four WeChat headlines containing *xiaojiejie*: two from popular media (PM) accounts, e.g., 自媒体 (self-media) and 营销账号 (marketing accounts), and two from state media (SM) accounts, e.g., 央视网 (CCTV Web) and 人民网 (People’s Web), respectively. The state media headlines were used as critical items and popular media headlines were used as fillers. The selection of the SM headlines was informed by the corpus-based analysis of the distinctive features of the two media sources. In particular, headlines that exemplify the use of *xiaojiejie* in direct address together with the second person pronoun and one additional lexical or grammatical feature and are therefore most representative of the headline style in state media were selected. The four judgment tasks appeared in randomized order.

Here is a sample question about a *xiaojiejie* headline from the state media account CCTV Web, translated into English:

When you see this headline [小姐姐! 你太太太太太太可爱啦!], which type of public account do you think it most likely comes from?
A. State media account
B. Self-media account
C. Marketing account
D. Other (please specify)

Hypothesis III predicts that respondents will select B, C, or D, but not A. In analyzing the responses, we quantified judgments of SM vs. PM (including self-media and marketing accounts) media source of headlines and compared these to the actual media source of the headlines.

The survey link was shared with four Chinese university professors who shared the link with their students. A total of 512 responses were received, of which 482 were complete. Of the respondents who completed the survey 92.3% fell in the 18–25 age group. Because this age group is famous for being the first generation of “digital natives” growing up constantly exposed to pop cultural trends and perpetually immersed in the new media, we selected this age group (*N*=445) for further analysis. Of these respondents, 0% said they “do not use WeChat” and 11.4% said they “occasionally use WeChat” and we eliminated them from further analysis. Of the remaining (*N*=394) respondents, 15.2% stated that they “rarely visit WeChat public accounts” and they were removed from further analysis. Of the remaining 334 respondents only four said they “have not heard of the address term *xiaojiejie*” and they were removed from the analysis. The remaining (*N*=330) responses (78.5% from female, 20.3% from male, and 1.2% from gender undisclosed respondents) made up the database for the analysis.

The survey research was part of a larger Chinese linguistics project, which was reviewed by the University of Oregon Research Compliance Services (RCS) under the 2018 Common Rule and determined to qualify for exemption under Title 45 CFR 46.104(d)((2)(i) Tests, surveys, interviews, or observation (non-identifiable)). To obtain informed consent, we included a privacy and information notice at the start of the survey, followed by a mandatory consent question (See S1 Appendix).

## Results

### Results on Baidu TGI data

To test the association between age and keyword, a chi-square of independence was computed on the TGI scores for *xiaojiejie* and *meinü* across five age groups obtained from Baidu Index (Table 1). There was a statistically significant association (*χ*^2^ = 50.178, *df* = 4, *p* < 0.0001) rejecting the null hypothesis that age and keyword are unrelated. The two younger and the two older groups showed opposite tendencies in terms of search interest in the keywords. The adjusted Pearson residuals show that the two younger and the two older age groups, but not the 30–39 group, made significant contributions to the chi-square statistic.

**Table 1:** TGI for xiaojiejie and meinü across five age groups.

Age	TGI		Adjusted residuals	
	*xiaojiejie*	*meinü*	*xiaojiejie*	*meinü*
< 19	103.26	56.6	**3.81**	**-3.81**
20–29	89.54	35.6	**4.97**	**-4.97**
30–39	131.45	149.99	-1.71	1.71
40–49	79.87	118.48	**-3.40**	**3.40**
> 50	58.62	85.08	**-2.65**	**2.65**

These results suggest that *meinü* is losing ground to *xiaojiejie* in popularity among members of the younger generation but remains popular among the older generation, confirming Hypothesis I that *meinü* is losing ground to *xiaojiejie* among younger Chinese.

### Results on headlines in WeChat public accounts

**Fig 2:**
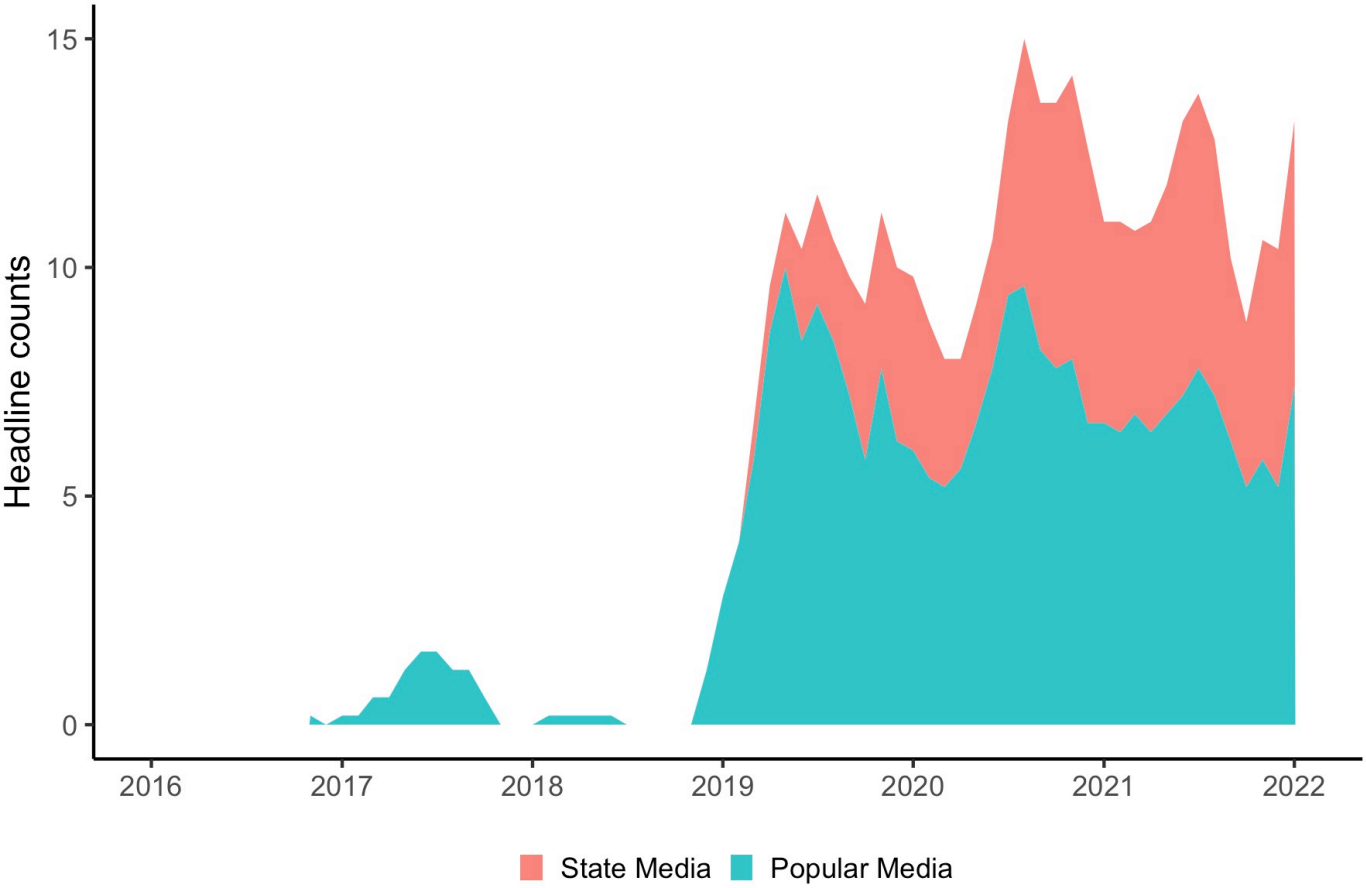
Frequency trends of xiaojiejie in headlines from two types of WeChat public accounts in a smoothed plot using five-month rolling averages.

The keyword search for *xiaojiejie* in WeChat public accounts, conducted in January 2022, returned a total of 437 headlines containing *xiaojiejie* the earliest instances of which can be traced back to 2016. The manual annotation process identified 287 headlines from popular or non-official media accounts and 150 headlines from official state media accounts. Fig 2 shows in a stacked area graph the frequency trends of *xiaojiejie* in headlines generated by the two types of WeChat public accounts at annual intervals spanning six years.

As can be seen in Fig 2, *xiaojiejie* headlines first occurred in WeChat popular media accounts in late 2016. Their frequencies remained low for more than two years until a sharp uptick took place in late 2018. No *xiaojiejie* headlines occurred in WeChat state media accounts up to this point. Starting in 2019, *xiaojiejie* headlines began to show up in state media accounts, though with low frequencies. Following a steep curve of “catch-up”, the frequency trajectories of *xiaojiejie* headlines in state media accounts converged with those of the popular media uses. These results show that *xiaojiejie* has spread from popular media to state media, confirming Hypothesis II.

To the extent that state media appropriated and caught up to popular media usage of *xiaojiejie*, the bottom-up spread of the address term can be considered lexical convergence. While *xiaojiejie* used in headlines can help both types of media grab attention, it remains to be seen what the headlines are about and what purposes they serve across media types. Comparing the lexical semantic, stylistic, and thematic features of the *xiaojiejie* headlines in the state vs. popular media corpora, we found that the *xiaojiejie* headlines in state media differed from those in popular media both formally and thematically and that they served the overarching goal of what China observers call “the ideological discourse of positive energy” [46].

Table 2 compares the 20 most frequently used signs in the *xiaojiejie* headlines from state media (on the left side) and the top 20 most frequently used items in those from popular media (on the right side) ranked by token frequency. To determine distinctive linguistic features across media types, we conducted unpaired two-samples Wilcoxon test, a non-parametric statistical method for comparing independent groups of samples, using the raw frequency of each sign in each corpus. The test identified positive keywords in each of the two corpora, namely items that are used with significantly greater frequency in one corpus than in the other and therefore distinguish one from the other [47].

**Table 2:** Top 20 most frequently used signs in headlines by state media vs. popular media.

State Media		Popular Media	
item	*p*-value	item	*p*-value
!	.000***	面膜 ‘sheet mask’	.002**
你 ‘you’	.039*	首 ‘CLF for songs’	.021*
网友 ‘netizen’	.000***	演唱 ‘sing’	.015*
铁路 ‘railway’	.000***	歌/歌曲 ‘song’	.022*
高铁 ‘high-speed train’	.000***	深情 ‘deep affection’	.022*
点赞 ‘like’	.002**	吧 *ba* (UFP)	.080
啦 *la* (UFP)	.006**	用 ‘use’	.313
样子 ‘appearance’	.001**	穿搭 ‘outfit’	.374
真美 ‘truly beautiful’	.003**	推荐 ‘recommendation’	.063
陌生 ‘strange’	.003**	经典 ‘classic’	.063
找到 ‘find’	.016*	帅 ‘handsome’	.874
白衣 ‘white dress’	.008**	腿 ‘leg’	.063
警员/民警/辅警 ‘police’	.002**	居然 ‘unexpectedly’	.063
呵斥 ‘scold’	.022**	歌声 ‘singing (voice)’	.130
青春 ‘youth’	.022*	歌手 ‘singer’	.130
救人 ‘save, rescue’	.022*	气质 ‘demeanor, vibe’	.130
治愈 ‘heal’	.022*	肤白 ‘light complexion’	.130
街头 ‘street’	.022*	好听 ‘pleasant to listen to’	.130
老人 ‘elderly person’	.022*	身材 ‘body figure’	.190
最美 ‘most beautiful’	.107	迷人 ‘attractive’	.190

As can be seen in Table 2, 19 out of the top 20 items in the corpus of state media headlines are positive keywords with statistical significance, some with strong statistical significance. The number of positive keywords in the popular media corpus is much smaller. Only five items show any statistical significance.

Among the positive keywords used in the *xiaojiejie* headlines by state media accounts are those that serve important communicative and performative functions, described as follows:

i) Signal enthusiasm, exuberance, and utter amazement

- Exclamation mark (!)
- Exclamative utterance final particle (UFP) 啦 la

ii) Construct an informal and endearing speech style

- Second person pronoun 你 ni ‘you’ directly addressing the referent of *xiaojiejie*
- Exclamative utterance final particle 啦 la

Consider the headlines in (1)-(3) from the WeChat public accounts of China’s top state media outlets.

(1) 小姐姐, 你太太太太太太可爱啦! (央视网 CCTV Web 6/28/2020)
  *Xiaojiejie*, ni tai tai tai tai tai tai ke-ai la
  Xiaojiejie, 2SG too too too too too too cute/lovable SFP
  ‘Xiaojiejie, you are toooooo cute!’
(2) 白衣小姐姐, 你溜走的样子真好看! (央视新闻 CCTV News 8/8/2020)
  Bai-yi *xiaojiejie*, ni liuzou-de yangzi zhen haokan
  White-dress xiaojiejie, 2SG run-away-ASSOC appearance really pretty
  ‘Xiaojiejie in white dress, the way you hurry away looks so pretty!’
(3) 小姐姐, 你飞奔的样子真美! (人民网 People’s Web 12/1/2021)
  *Xiaojiejie*, ni fei-ben-de yangzi zhen mei
  Xiaojiejie, 2SG fly-run-ASSOC appearance really beautiful
  ‘Xiaojiejie, the way you dash looks so beautiful!’

These exclamative headlines are performative and socially indexical in the sense that they perform a semiotic act using supposedly pop-cultural linguistic devices for the display and construction of a pop cultural identity in interaction [48, 49]. Their style is so jaunty and jolly that it embodies nothing of the serious and stern tone of party discourse in the pre-digital age, as characterized by Li [50]. The headlines are vividly gripping but vacuous in terms of news content. They do not disclose who and what they refer to until the reader clicks on them to discover the stories. Thus, the headlines are designed, first and foremost, to attract clickbait, in the same way as *meinü* [29]. As each of the stories unfolds with a click, the reader discovers that the person called *xiaojiejie* in (1) was a young and dedicated staff member of a local CDC in Beijing who worked tirelessly on night shifts during the Covid-19 pandemic so much so that she had no time to sleep or eat regular meals and gained weight she said she was too busy to shed. The *xiaojiejie* in example (2) was an anonymous and altruistic young woman who hurried away after secretly paying for the night snacks for a group of firefighters. The one addressed as *xiaojiejie* in (3) was a young woman who ran to the rescue of a little boy lost in the middle of a dangerously busy thoroughfare. Hailing these young women as beautiful heroes and lovable paragons, these headlines illustrate the energetic Foucauldian uses of linguistic signs by the state media for the purpose of propaganda [46]. As can be seen in the positive keywords shown in Table 2, railway employees and police officers are frequently featured in these positive-energy stories.

By contrast, the *xiaojiejie* headlines in the popular media accounts are formally diverse, as no grammatical morpheme is preferred with statistical significance. They are thematically mundane, focusing on beauty, fashion, entertainment and fitness, with only a few positive keywords recurring at statistically significant levels, such as ‘sheet masks’ and ‘singing’. In other words, *xiaojiejie* headlines in the popular media are structurally less marked and are not engineered for the same overarching propagandistic purpose as seen in the state media headlines. The differences between the two corpora measured by positive keywords suggest that the state media accounts are perhaps trying too hard in crafting an informal, cutesy, and supposedly popular voice, resulting in a netspeak overkill in their *xiaojiejie* headlines. However, as our survey results show, Chinese WeChat users could not distinguish such tryhard headlines from those posted by popular media, to which we now turn.

### Results from the Qualtrics survey

**Fig 3:**
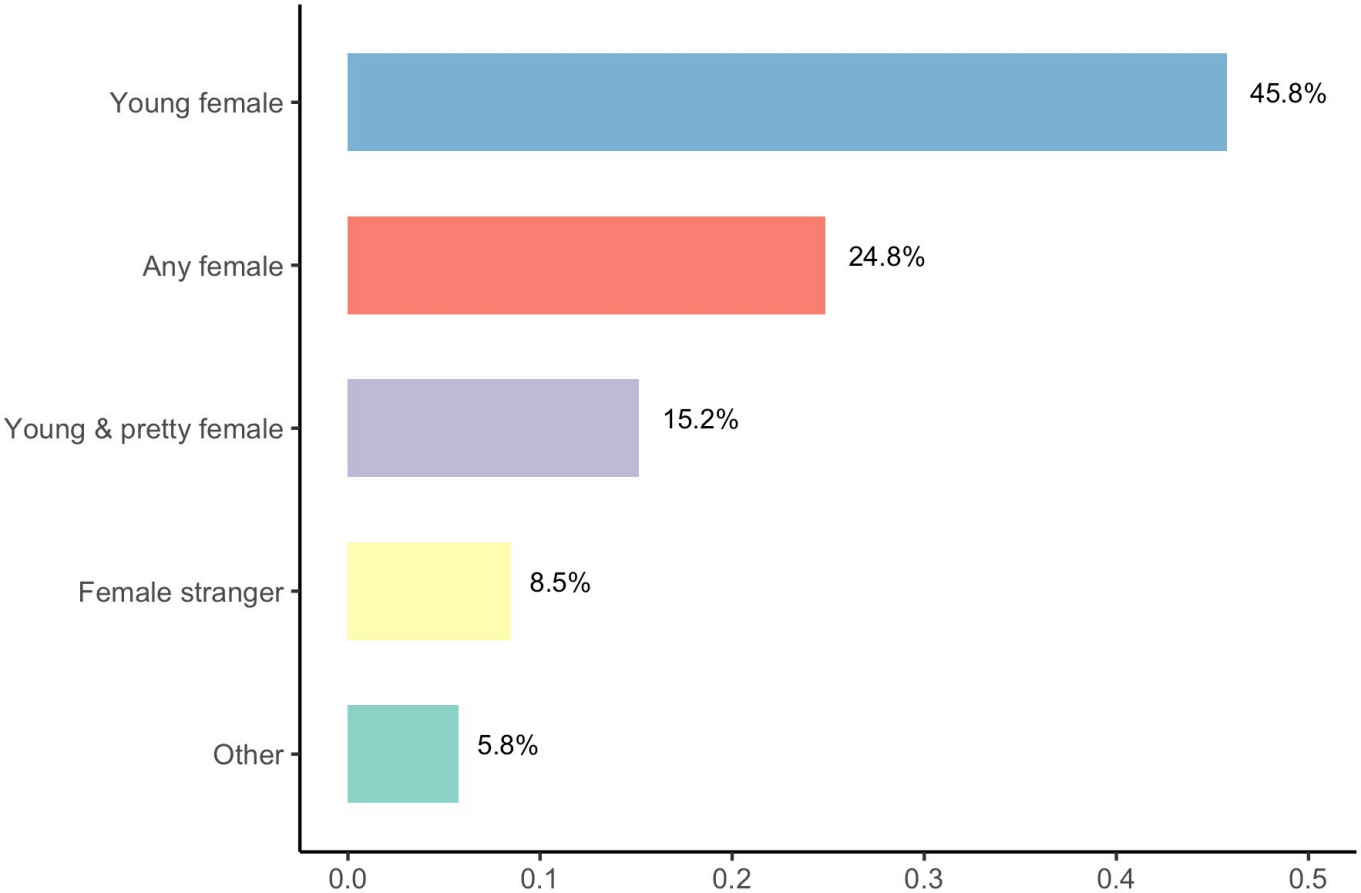
Interpretations of who xiaojiejie is used to address or refer to.

So far we have taken for granted that *xiaojiejie* is a social address without delimiting its referential scope. In testing Hypothesis III, our survey wanted first to clarify the addressee or referent of *xiaojiejie* by asking an open-ended question as to who the respondents think *xiaojiejie* is used to address or refer to. We sorted the 330 responses into four groups using the bucketing function in Qualtrics and the results are shown in Fig 3.

Fig 3 suggests disagreements on the role played by age, looks, and familiarity of the addressee in the respondents’ understanding of who the term addresses or refers to. The disagreements notwithstanding, there is overwhelming agreement that *xiaojiejie* is a social address most commonly used to address or refer to young women and is possibly undergoing a referential extension to women in general.

**Fig 4:**
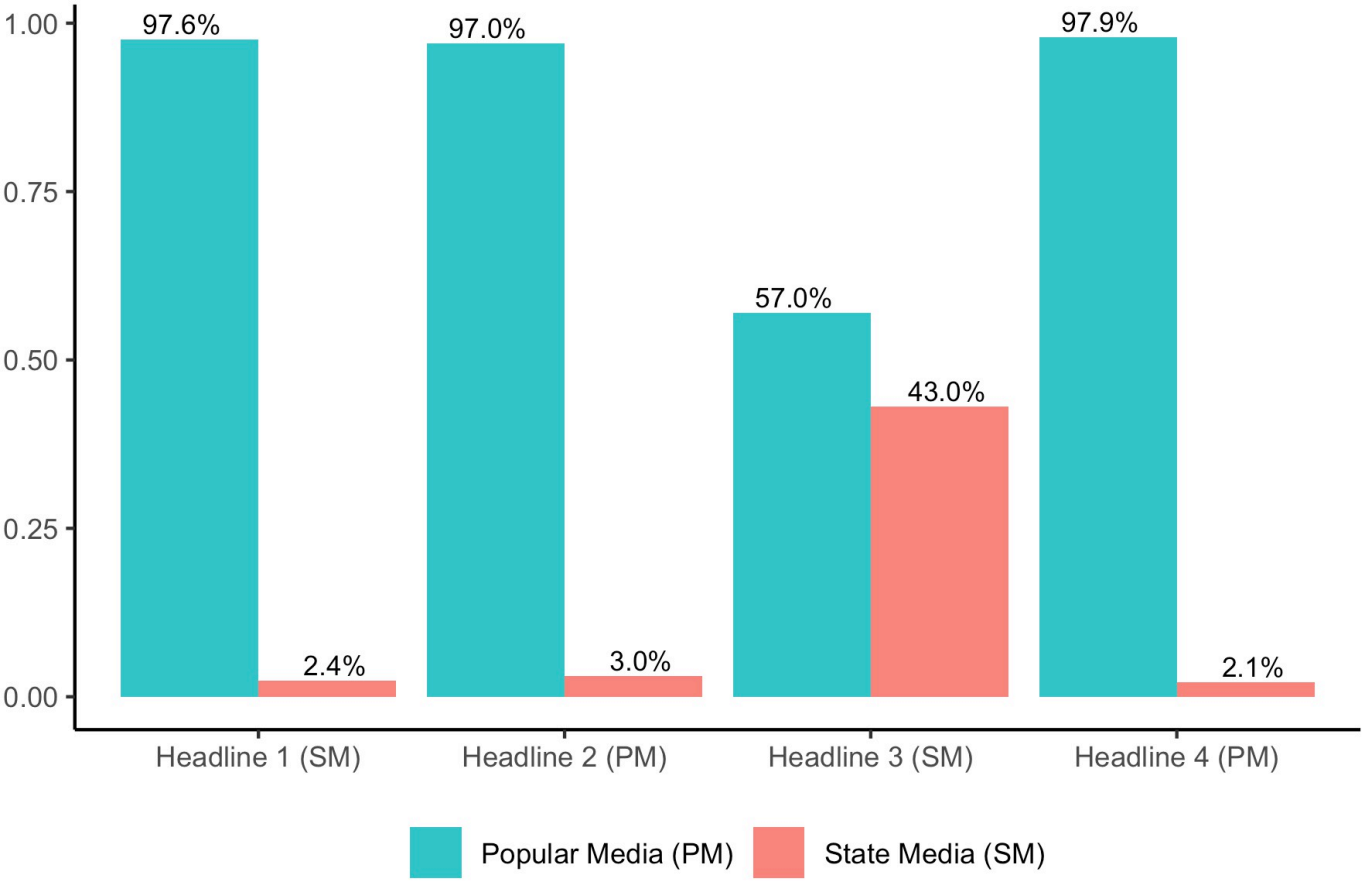
Judgments on media source of xiaojiejie headlines from state media (SM) vs. popular media (PM).

The results of the judgment tasks show that the web-savvy respondents could not tell headlines generated by the state media (SM) public accounts from those by the popular media (PM) accounts. As shown in Fig 4, respondents correctly judged the popular source of the two *xiaojiejie* headlines posted by the popular media (PM) accounts; but they misjudged the two *xiaojiejie* headlines from SM accounts as the products of popular media (PM). Nearly 50% of the respondents judged the the headline from *CCTV Web* (小姐姐, 你太太太太太太可爱啦! ‘*Xiaojiejie*, you are toooooo cute!’) as coming from a marketing type of popular public account. As for the headline from *People’s Web* (小姐姐, 你飞奔的样子真美! ‘*Xiaojiejie*, the way you dash looks so beautiful!’), approximately one third of the respondents judged it as coming from a marketing account and almost a quarter of the respondents judged it as coming from a self-media account. These results demonstrate that the blurry linguistic boundary between popular and state media in the usage of *xiaojiejie* is perceptually real, confirming Hypothesis III.

## Discussion and conclusion

Filling the gap in existing research on China’s convergence culture, our study has provided sociolinguistic evidence of the blurry lines between popular and institutional media. Our analysis of Baidu TGI by age showed that the previously viral social address *meinü* no longer holds the attention of the younger generations who pay more attention to *xiaojiejie*, a cutesy new address term with an origin in teenage fandom, confirming Hypothesis I: *meinü* is losing ground to *xiaojiejie* among younger Chinese. Our analysis of the *xiaojiejie* headlines in WeChat public accounts showed that *xiaojiejie* was first used by popular media accounts when the fandom-based uses expanded from their original context. State media accounts adopted the novel address term upon the visible surge of the popular usage and continued to follow and catch up with the linguistic trends in popular media, confirming Hypothesis II: *xiaojiejie* has spread from popular media to state media in what can be considered lexical and stylistic convergence. Our online survey results showed that young and regular Chinese WeChat users who invariably claimed they knew the term *xiaojiejie* misidentified the source of *xiaojiejie* headlines produced by state media, judging them as originating in popular media despite the fact that these headlines bear the typical linguistic stamps of state media accounts that are not found in popular media accounts. The findings confirmed Hypothesis III: The blurring boundaries between popular and state media in their uses of *xiaojiejie* have a perceptual reality.

Jenkins [4] described convergence culture as a participatory culture. Social address offers a linguistic lens on China’s convergence culture [29]. The findings from the present study are consistent with the previous insight that state digital media eagerly participates in popular language use for the purpose of propaganda. Not only is state media sensitive to the waxing and waning of popular address terms in the netspeak marketplace, it tries hard to keep up with the ever shifting popular linguistic trends. Form follows function. In the digital space of media convergence, the perky and personalizing style of the *xiaojiejie* headlines indexical of a popular media persona is constructed by state media as an adaptive vehicle of party propaganda to drive clickbait and to remain relevant in the digital mediascape. This finding is consistent with available insights from media studies conducted elsewhere that suggest conventional mainstream media adopt alternative media strategies to stay relevant and relatable [38].

More significantly, our perceptual study is the first to provide empirical evidence that the uses of the popular social address by state media have effectively blurred the linguistic lines between popular culture and party propaganda. Linguistic innovations happen all the time. Throughout Chinese history, major waves of lexical innovations occurred at important sociocultural junctures. However, as Jing-Schmidt and Hsieh [51] stated, Chinese Internet neologisms distinguish themselves from earlier lexical innovations initiated by cultural and intellectual elites and ideological authorities. They are created by grassroots and spread horizontally in networked communication and some usages ultimately spread upwards to mainstream media and even enter “the calculated vocabulary of politicians”. In the age of the attention economy under media convergence in a platform society, performative participation drives the linguistic enmeshment among platformed media stakeholders and blurs the formal boundaries between institutional and popular media products. Our finding that young Chinese WeChat users who are “digital natives” were unable to distinguish state media headlines from popular media headlines containing *xiaojiejie* shows that the blurry linguistic boundaries between popular media and party propaganda are perceptually real. The young users’ confusion sheds light on the extent and cognitive effects of media convergence and raises questions about the consequences of convergence culture for political cognition.

Finally, in concluding this article we wish to draw methodological implications for linguistic research on convergence culture. Combining platform-based big data on user interest, time series data of usage trends, contrastive corpus analysis of lexical grammatical patterns, and sociolinguistic survey analysis of user perception, our study has demonstrated the utility of complementary data and methods in answering research questions and hopefully points to new terrains of research and knowledge production in the study of language in convergence culture.

## Supporting information

S1 Appendix Survey. (PDF)
