# Supplementary material for: The blurry lines between popular media and party propaganda: China’s convergence culture through a linguistic lens
Source: PLoS One. 2024 Jan 25;19(1):e0297499. doi: 10.1371/journal.pone.0297499 (PMC10810527; doi:10.1371/journal.pone.0297499)
Supplement: S1 Appendix — (PDF) [file pone.0297499.s001.pdf]

Appendix: Survey

CHINESE ORIGINAL

欢迎参加本研究调查!

您好！本研究的目的是调查汉语称呼语的使用。请您回答几个与此有关的问题。问卷完全匿名，请您放心。此调查只需2-3分钟即可完成。您参与此项研究纯属自愿，并有权因任何原因在调查期间随时退出，且不会对您产生任何偏见。如果您想联系研究负责人讨论此研究，请发送电子邮件至 xxx@xxx。您的母语是汉语，您已满18周岁，且了解您可以因任何原因随时终止参与此调查。点击同意按钮即表示您自愿参与此调查。

o 同意，开始调查
o 不同意，拒绝参加

请问您的性别是?
o 女性
o 男性
o 不方便说

请问您的年龄?
o 18-25 岁
o 26岁及以上

您使用微信吗?
o 天天用、离不开
o 偶尔使用

您看微信公众号吗?
o 经常看

o 偶尔看
o 很少看

您听说过小姐姐这个说法吗?
o 听说过
o 没听说过

如果听说过【小姐姐】这个说法，您认为这个称呼是指谁? (请在空格填写)

____

(Block with question randomization)

假如在微信公众号看到【小姐姐！你太太太太太太可爱啦！】这个标题，您觉得它最有可能来自哪一类公众号?
o 营销公众号
o 官媒公众号
o 自媒体公众号
o 其他(请简要说明) ________________

假如在微信公众号看到【高颜值的小姐姐为什么都开始跑步?】这一标题，您觉得它最可能来自哪一类公众号?
o 营销公众号
o 官媒公众号
o 自媒体公众号
o 其他(请简要说明) ________________

假如在微信公众号看到【小姐姐，你飞奔的样子真美！】这一标题，您觉得它最可能来自哪一类公众号?
o 营销公众号
o 官媒公众号

o 自媒体公众号
o 其他(请简要说明) ________________

假如在微信公众号看到【157斤胖阿姨，成功变身小姐姐！】这一标题，您觉得它最可能来自哪一类公众号？
o 营销公众号
o 官媒公众号
o 自媒体公众号
o 其他(请简要说明) ________________

提交

非常感谢您在百忙之中抽出时间填写问卷！

ENGLISH TRANSLATION

Welcome to participate in this research survey!

Hello! The purpose of this study is to investigate the usage of Chinese address terms. This questionnaire will ask you some related questions. Please be assured that your response will be completely confidential. It takes about 2 to 3 minutes to complete the survey. Participation in this study is purely voluntary. You have the right to exit the survey at any time for any reason during the course of this research and will not be subjected to any prejudice. If you would like to contact the person in charge of the study to discuss the study, please send an email to xxx@xxx. Your native language is Chinese, you are over 18 years old, and you understand that you can terminate your participation in this survey at any time for any reason. Clicking the "Agree" button indicates your voluntary participation in this survey.

o Agree, start the survey
o Disagree, refuse to participate

What is your gender?
o Female
o Male
o Prefer not to say

What is your age?
o 18-25 years old
o 26 years old and above

How often do you use WeChat?
o Every day
o Occasionally

How often do you read articles from WeChat Official Accounts?
o Often
o Occasionally
o Rarely

Have you heard of the term "小姐姐" (xiaojiejie)?
o Yes
o No

If you've heard of the term "小姐姐" (xiaojiejie), who do you think this term refers to? (Please fill in the blank)

____

(Block with question randomization)

If you encounter the title "小姐姐！你太太太太太太可爱啦！" on a WeChat Official Account, which type of account do you think it's most likely from?
o Marketing Official Account
o Official Media Account
o Self-Media Account
o Other (Please briefly explain) ___________________

If you encounter the title "高颜值的小姐姐为什么都开始跑步？" on a WeChat Official Account, which type of account do you think it's most likely from?
o Marketing Official Account
o Official Media Account
o Self-Media Account
o Other (Please briefly explain) _________________

If you encounter the title "小姐姐，你飞奔的样子真美！" on a WeChat Official Account, which type of account do you think it's most likely from?
o Marketing Official Account
o Official Media Account
o Self-Media Account
o Other (Please briefly explain) _________________

If you encounter the title "157斤胖阿姨，成功变身小姐姐！" on a WeChat Official Account, which type of account do you think it's most likely from?
o Marketing Official Account
o Official Media Account
o Self-Media Account
o Other (Please briefly explain) _________________

Submit

Thank you very much for taking the time to fill out the questionnaire in your busy schedule!
